# Long Noncoding RNA MALAT-1 Enhances Stem Cell-Like Phenotypes in Pancreatic Cancer Cells

**DOI:** 10.3390/ijms16046677

**Published:** 2015-03-24

**Authors:** Feng Jiao, Hai Hu, Ting Han, Cuncun Yuan, Lei Wang, Ziliang Jin, Zhen Guo, Liwei Wang

**Affiliations:** 1 Department of Medical Oncology and Pancreatic Cancer Center, Shanghai General Hospital, Shanghai Jiao Tong University School of Medicine, Shanghai 201620, China; E-Mails: jiao_f@outlook.com (F.J.); huhai87@126.com (H.H.); yyhanwh@163.com (T.H.); wang_lei744@hotmail.com (L.W.); 2 Shanghai Key Laboratory of Pancreatic Diseases, Shanghai 201620, China; E-Mails: jzl920222@126.com (Z.J.); guozhenzhen@126.com (Z.G.); 3 Department of Pathology, Shanghai General Hospital, Shanghai Jiao Tong University School of Medicine, Shanghai 201620, China; E-Mail: blkcun@126.com

**Keywords:** MALAT-1, stemness, long noncoding RNA, cancer stem cells

## Abstract

Cancer stem cells (CSCs) play a vital role in tumor initiation, progression, metastasis, chemoresistance, and recurrence. The mechanisms that maintain the stemness of these cells remain largely unknown. Our previous study indicated that MALAT-1 may serve as an oncogenic long noncoding RNA in pancreatic cancer by promoting epithelial-mesenchymal transition (EMT) and regulating CSCs markers expression. More significantly, there is emerging evidence that the EMT process may give rise to CSCs, or at least cells with stem cell-like properties. Therefore, we hypothesized that MALAT-1 might enhance stem cell-like phenotypes in pancreatic cancer cells. In this study, our data showed that MALAT-1 could increase the proportion of pancreatic CSCs, maintain self-renewing capacity, decrease the chemosensitivity to anticancer drugs, and accelerate tumor angiogenesis *in vitro*. In addition, subcutaneous nude mouse xenografts revealed that MALAT-1 could promote tumorigenicity of pancreatic cancer cells *in vivo*. The underlying mechanisms may involve in increased expression of self-renewal related factors Sox2. Collectively, we for the first time found the potential effects of MALAT-1 on the stem cell-like phenotypes in pancreatic cancer cells, suggesting a novel role of MALAT-1 in tumor stemness, which remains to be fully elucidated.

## 1. Introduction

Pancreatic cancer is one of the most aggressive and lethal malignancies, with estimated 46,420 new pancreatic cancer cases and 39,590 cancer deaths in the United States in 2014 [1]. It is projected to become the second most common cause of cancer deaths in the United States by 2030 [2]. Despite remarkable progress in understanding pancreatic carcinogenesis at the molecular level, as well as progress in new therapeutic approaches, pancreatic cancer remains a disease with a dismal prognosis. Emerging evidence has suggested that the presence of a small subset of cells, termed cancer stem cells (CSCs) in pancreas tumors contributes to cell growth, drug resistance, invasion, metastasis and recurrence [3].

Long non-coding RNAs (lncRNAs) are defined as endogenous cellular RNAs more than 200 nucleotides in length that lack an open reading frame of significant length [4]. In recent years, several lncRNAs have been shown to be involved in carcinogenesis and cancer progression [4]. Metastasis-associated lung adenocarcinoma transcript 1 (MALAT-1), an evolutionarily highly conserved and ubiquitously expressed lncRNA, was found to be highly overexpressed in several human malignancies, and associated with clinical parameters and promoted tumor cell invasion and metastasis [5]. The underlying mechanisms might involve in inducing epithelial-mesenchymal transition (EMT) by regulating the expression of relevant genes [6,7]. Moreover, accumulating evidences suggest that cells can acquire stem-like properties during induction of EMT [8,9]. Several studies have reported that pancreatic CSCs also possess mesenchymal features [10,11,12]. Our previous study indicated that MALAT-1 may serve as an oncogenic lncRNA that is involved in malignancy phenotypes of pancreatic cancer, and revealed that MALAT-1 knockdown could suppress EMT process and decrease the protein expression of cancer stem-like cell markers including CD44, CD24 and ALDH [13]. Therefore, we hypothesize that MALAT-1 could enhance stem-like phenotypes in pancreatic cancer cells. In this study, we provide multiple lines of evidence for the crucial role of MALAT-1 on pancreatic CSC-like properties.

## 2. Results

### 2.1. MALAT-1 Was Upregulated in CSCs and Could Increase the Proportion of CSCs in Pancreatic Cancer Cells

**Figure 1:**
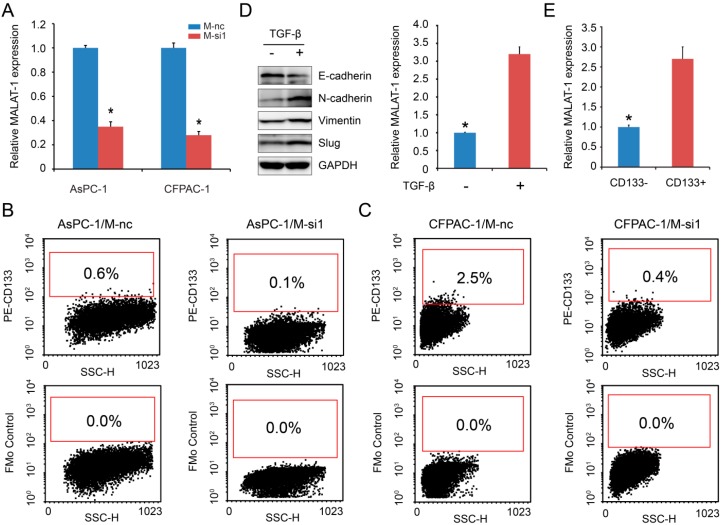
Metastasis-associated lung adenocarcinoma transcript 1 (MALAT-1) was upregulated in cancer stem cells (CSCs) and could increase the proportion of CSCs in pancreatic cancer cells. (**A**) Relative MALAT-1 expression was detected using real-time-quantitative polymerase chain reaction (RT-qPCR); (**B**,**C**) Flow cytometric analysis was used to determine the CD133 expression on the cell surface of pancreatic cancer cell lines AsPC-1/M-nc and AsPC-1/M-si1 (**B**), as well as CFPAC-1/M-nc and CFPAC-1/M-si1 (**C**). The percentage of CD133^+^ subpopulation was decreased following MALAT-1 downregulation; (**D**) EMT-induced cell by treatment with TGF-β had higher MALAT-1 expression compared to that of control; (**E**) Higher level of MALAL-1 expressed in CD133^+^ cell. Data are shown as mean ± SD. * *p* < 0.05 compared with the control group.

Pancreatic cancer cell lines AsPC-1/M-si1 and CFPAC-1/M-si1, which stably knock down expression levels of MALAT-1, as well as that of controls (AsPC-1/M-nc and CFPAC-1/M-nc) have been established in our previously study [13]. To further illustrate the role of MALAT-1 in pancreatic CSC-like properties, we firstly assessed the knockdown effect by RT-qPCR, and the results confirmed that M-si1 could achieve the great efficacy in silencing MALAT-1 expression compared to the negative control M-nc (Figure 1A). Next, we detected altered expression of CD133, a putative CSCs marker in pancreatic tumors [14], following MALAT-1 downregulation by flow cytometry. The results showed that the percentage of CD133^+^ subpopulation decreased from 0.6% (AsPC-1/M-nc) to 0.1% (AsPC-1/M-si1), 2.5% (CFPAC-1/M-nc) to 0.4% (CFPAC-1/M-si1) respectively (Figure 1B,C). In addition, TGF-β treatment for a period of 48 h induced EMT in CFPAC-1 cell line, as reflected by the altered expression of the EMT-marker genes E-cadherin, Vimentin, and *N*-cadherin at protein level (Figure 1D). We found that EMT-induced pancreatic cancer cells had higher MALAT-1 expression compared to that of control (Figure 1D). Furthermore, we compared MALAT-1 expression between CD133^+^ and CD133^−^ CFPAC-1 cells, which were sorted by flow cytometry. Figure 1E showed higher expression level of MALAL-1 in CD133^+^ cell. The above data indicated that MALAT-1 was upregulated in CSCs and could increase the proportion of CSCs in pancreatic cancer cells.

### 2.2. MALAT-1 Enhances Spheroid Forming Ability and Anchorage Independent Growth of Pancreatic Cancer Cells

**Figure 2:**
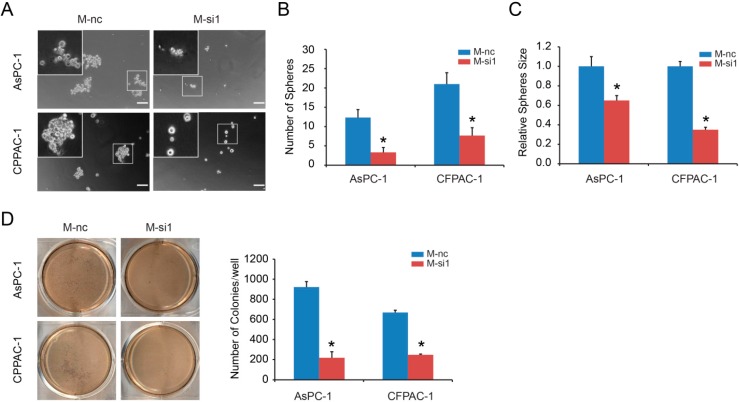
MALAT-1 enhanced spheroid forming ability and anchorage independent growth in pancreatic cancer cells. The capacity of sphere formation (Scale bar, 50 μm) (**A**–**C**) were compared between M-nc and M-si1 groups. MALAT-1 knockdown reduced the number (**B**) and size (**C**) of tumor spheres. Anchorage independent growth showed that M-si1 cells had the decreased ability to form colonies in soft agar (**D**). Data are shown as mean ± SD. * *p* < 0.05 compared with the control group.

Next, we used *in vitro* sphere formation assay to examine whether MALAT-1 participates in CSC renewal. The results showed that MALAT-1 knockdown reduced the formation of spheres significantly, and the size of tumor spheres in M-si1 groups was significantly smaller than that of M-nc groups (Figure 2A–C). Further, anchorage independent growth exhibited that M-si1 cells had the decreased ability to form colonies in soft agar (Figure 2D). These experiments implicate that MALAT-1 has an important role in the regulation of pancreatic CSCs and is necessary for the self-renewing capacity.

### 2.3. MALAT-1 Decreases Chemosensitivity of Gemcitabine in Pancreatic Cancer Cells

**Figure 3:**
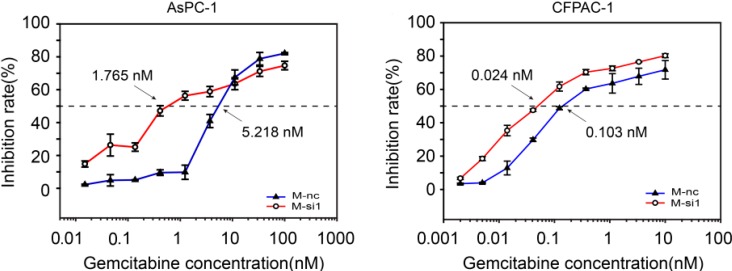
Elevated level of MALAT-1 decreases chemosensitivity of gemcitabine in pancreatic cancer cells. M-nc and M-si1 cells were exposed to gemcitabine at different concentrations. A 50% inhibitory drug concentration (IC_50_) of gemcitabine was significantly higher in M-nc groups in comparisons with M-si1 groups. Data are shown as mean ± SD.

Resistance to chemotherapy is another property that can distinguish pancreatic CSCs from other cancer cells [15]. We therefore investigated the impact of candidate drugs gemcitabine, a commonly used anti-cancer agent against pancreatic carcinoma in clinic, on the cell proliferation, and calculated the 50% inhibitory drug concentration (IC_50_) following MALAT-1 knockdown. Figure 3 showed the antiproliferative effects of gemcitabine in M-nc and M-si1 cells. The IC_50_ value of gemcitabine in AsPC-1/M-nc and CFPAC-1/M-nc was 5.218 and 0.103 nM, respectively, whereas that in M-si1 cells was 1.765 and 0.024 nM, respectively. The resistance index (RI) [16] was determined as the ratio of the IC50 of the M-nc cells *vs.* the IC_50_ of M-si1. The RI of gemcitabine in AsPC-1/M-nc and CFPAC-1/M-nc was 2.96 and 4.29 times higher than that of M-si group, respectively. The above date suggested that elevated level of MALAT-1 could decrease chemosensitivity of gemcitabine in pancreatic cancer cells.

### 2.4. Elevated Expression Levels of MALAT-1 in Pancreatic Cancer Cells Accelerate HUVEC Tube Formation and Migration

**Figure 4:**
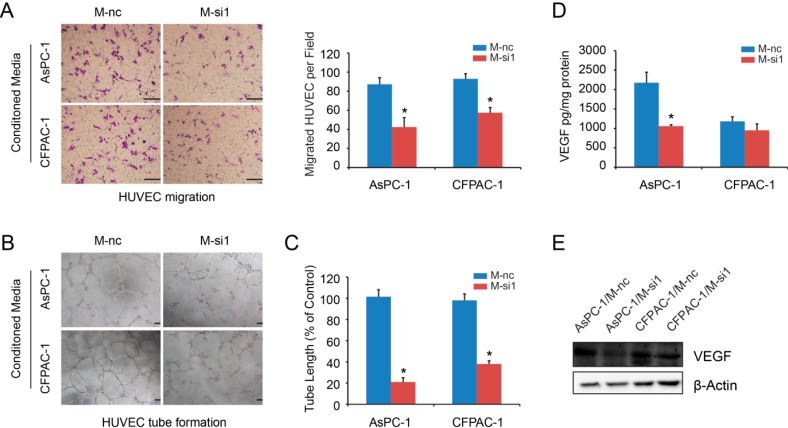
Elevated expression levels of MALAT-1 in pancreatic cancer cells induced endothelial cell migration and tube formation. (**A**) Transwell migration assay showed that conditioned media from M-si groups decreased HUVEC migration significantly compared to those from M-nc groups (Scale bar, 25 μm); (**B**,**C**) HUVEC tube formation assay. Significant decreases in the tube number (**B**) and tube length (**C**) were observed in conditioned media from M-si groups compared with those from M-nc groups (Scale bar, 200 μm); (**D**) VEGF concentration was detected by ELISA. ELISA values were corrected for total cell protein; (**E**) Western blotting analysis was employed to detect VEGF protein expression of cells lysate. Data are shown as mean ± SD. * *p* < 0.05 compared with the control group.

Accumulating evidence has shown that CSCs interact closely with angiogenesis [17]. We tested the ability of conditioned media from both M-nc and M-si1 cells to modify endothelial cell phenotypes. The results showed that conditioned medium from M-nc cell consistently increased migration of HUVEC as compared with those from M-si1 cells (Figure 4A). The addition of conditioned medium from M-nc cell cultures also promoted endothelial cell tube formation *in vitro* by increasing HUVEC tube length, number of branch points, and tube complexity (Figure 4B,C). Next, we compared VEGF concentration between M-nc and M-si1 groups by ELISA. The results showed that, in AsPC-1 cell, VEGF levels from M-nc group conditioned media were upregulated in comparisons with M-si1 media (Figure 4D). However, for CFPAC-1, the two groups had no statistically difference (Figure 4D). Another angiogenesis-related cytokines may participate in this pro-angiogenic function. Western blot analysis of cells lysate supported the ELISA results (Figure 4E). Taken together, these data provide strong evidence that MALAT-1 can induce angiogenesis *in vitro*.

### 2.5. MALAT-1 Promotes Tumorigenicity of Pancreatic Cancer Cells in Vivo 

**Figure 5:**
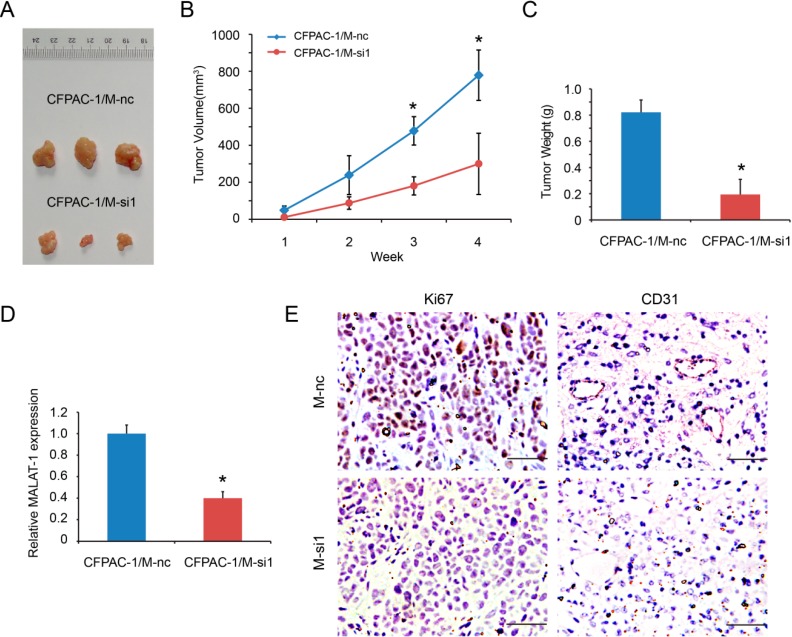
MALAT-1 knockdown reduced xenografts growth of CFPAC-1 cells. (**A**) Representative photographs of the subcutaneous tumors; (**B**) The sizes of the tumors developed in mice injected with CFPAC-1/M-nc (*n* = 3) were significantly larger than those that in mice injected with CFPAC-1/M-si1 (*n* = 3). Tumor volume was calculated following the formula of Volume (mm^3^) = (A × B^2^)/2, where A is the longest diameter of tumor and B is the shortest diameter; (**C**) The average tumor weight of xenografts was also lower in M-si group; (**D**) Xenografts were collected for RNA extraction and detected for MALAT-1 expression by RT-qPCR; (**E**) Expression of Ki67 and CD34 was detected by immunohistochemistry (Scale bar, 50 μm). Data are shown as mean ± SD. * *p* < 0.05 compared with the control group.

We finally examined whether MALAT-1 promoted the growth of pancreatic cancer cells *in vivo*, as the tumorigenic capacity *in vivo* is considered to a characteristic feature of CSCs [3]. The data showed that the growth rate of CFPAC-1/M-si1 xenografts was slower than that in control group (Figure 5A,B), and the average tumor weight of xenografts was also lower (0.19 ± 0.12 *vs.* 0.82 ± 0.09 g) (Figure 5C). In addition, xenografts were collected for RNA extraction and detected for MALAT-1 expression. The results showed that M-si1 group had lower MALAT-1 expression compared to that of M-nc group (Figure 5D), ensuring that the effect of MALAT-1 *in vivo*. To investigate MALAT-1 expression whether affect *in vivo* biological function, we detected Ki67 expression for tumor cell proliferation and CD31 for angiogenesis by immunohistochemistry. The results revealed that Ki67 and CD31 expression was significantly reduced in M-si1 group (Figure 5E). Together, the above data indicate that MALAT-1 enhances the tumorigenicity and stemness of pancreatic cancer cells *in vivo*.

### 2.6. Downregulation of MALAT-1 Reduces Self-Renewal Associated Factors Expression of Pancreatic Cancer Cells

**Figure 6:**
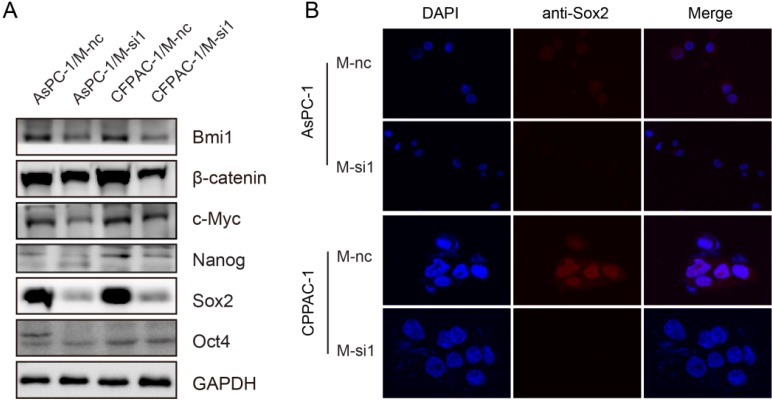
Knockdown of MALAT-1 reduces self-renewal associated factors expression of pancreatic cancer cells. (**A**) Expressions of Oct4, Nanog, Sox2, Bmi1, β-catenin and c-Myc proteins in M-nc and M-si1 cells were detected by Western blot; (**B**) The expression and subcellular localization of Sox2 were evaluated by immunofluorescence.

The most important and useful property of stem cells is that of self-renewal. Several pluripotency-related transcriptional factors and similar key signaling pathways may regulate self-renewal in stem cells and CSCs. To explore the underlying mechanisms of MALAT-1 in regulating CSCs-like properties, we examined several self-renewal related factors including Oct4, Nanog, Sox2, Bmi1, β-catenin and c-Myc that play pivotal roles in the maintenance of stem cells in response to the knockdown of MALAT-1. The results showed that Sox2 was significantly decreased in M-si1 compared with the M-nc groups, but Bmi1 and Nanog decreased a little, Oct4 not at all, c-Myc in only one cell line AsPC-1 (Figure 6A). Moreover, the expression and subcellular localization of Sox2 were evaluated by immunofluorescence. As shown in Figure 6B, the immunofluorescent images demonstrated higher levels of Sox2 in M-nc groups. The data together suggest that the enhanced stem cell-like phenotypes of MALAT-1 on pancreatic cancer possibly through upregulating the expression of self-renewal related factors Sox2. However, the detailed molecular mechanisms of MALAT-1 upregulating Sox2 require further exploration.

## 3. Discussion

According to the CSCs hypothesis, only a small fraction of cells, namely the CSCs, within a tumor is multipotent and has the capability of indefinite self-renewal and asymmetric cell division [18,19]. There is emerging evidence that CSCs might be playing an important role in cancer aggressiveness, metastasis, recurrence, resistance to chemotherapy and overall survival in hematologic malignancies as well as solid tumors including pancreatic adenocarcinoma [20]. MALAT-1 served as an oncogenic lncRNA has been linked to malignant phenotype in several human tumor entities [5]. And EMT plays a critical role in MALAT-1 mediated pancreatic cancer migration and invasion [13]. More significantly, there is emerging evidence that the EMT process may give rise to CSCs, or at least cells with stem cell-like properties [21]. Therefore, we hypothesize that MALAT-1 could enhance stem-like properties in pancreatic cancer cells.

Several cell-surface markers, including CXCR4 [22], ALDH [23], CD24 [24] and CD44 [25] have been used to identify of cancer stem-like cells in pancreatic cancer. We have previously performed western blot analysis to reveal that knockdown of MALAT-1 decreased the levels of CD44, CD24 and ALDH expression significantly [13]. Moreover, CD133, or prominin-1, has also been used to identify putative CSCs in pancreatic tumors [26]. Herein, we revealed that CD133 expression was reduced after MALAT-1 downregulation, and higher level of MALAL-1 was expressed in CD133^+^ cell. In addition, higher MALAT-1 expression was also found in TGF-β induced EMT cell. The above data suggested that MALAT-1 was upregulated in CSCs and could increase the proportion of CSCs in pancreatic cancer cells.

Maintenance of cell sphere formation capacity and the capability of anchorage independent growth have been used as read-out for tumor stemness [27,28]. Our data showed that MALAT-1 knockdown reduced cell sphere formation capacity and the capability of anchorage independent growth significantly *in vitro*. In addition, the nude mouse transplantation tumor experiment displayed that MALAT-1 enhanced the tumorigenicity pancreatic cancer cell *in vivo*. CSCs, also characterized by a high intrinsic resistance to chemo- and radio-therapy, will survive and give rise to a new generation of cancer cells that are resistant towards the applied chemotherapeutics [29]. A recently study showed that several genes involved in tumor development and metastasis were similarly induced in CSCs and cisplatin-resistant non-small-cell lung cancer cell lines H460 by gene expression analysis [30]. Interestingly, MALAT-1 was included in above genes [30]. Our result demonstrated that reduced MALAT-1 level could decrease IC_50_ of gemcitabine. However, the difference is not very different. Therefore, we cannot exclude possibility that the increased chemosensitivity is a reflection of the slower growth rate of the MALAT-1 knockdown. Collectively, the above data suggested that high level of MALAT-1 in pancreatic cancer cells could be endowed with stem cell-like properties. 

Next, our studies provide strong evidence that MALAT-1 can induce angiogenesis *in vitro*. Accumulating evidence has shown that CSCs interact closely with angiogenesis [17]. Tumor angiogenesis is induced by CSCs, expressing proangiogenic factors such as VEGF to the tumor microenvironment [31,32]. On the other hand, tumor angiogenesis and formation of a cancer vascular niche contribute to maintenance and further proliferation of CSCs [17,33]. Interestingly, a recent study revealed that the involvement of MALAT-1 in diabetes-induced microvascular dysfunction, and inhibition of MALAT-1 may serve as a novel therapeutic strategy for diabetes-related microvascular complications [34].

The mechanisms of MALAT-1 in regulating CSCs-like properties were unclear. Deregulation of several signaling molecules implicated in the self-renewal process of normal stem cells has been associated with stemness of cancer cells and tumorigenesis [35]. Several transcription factors, including Oct4, Nanog and Sox2, are aberrantly expressed in pancreatic cancer and contribute to pancreatic CSC-like characteristics [36]. Several genes that are frequently upregulated in tumors, such as Bmi1 [37,38] c-Myc [39,40] and β-catenin [41,42], have been shown to contribute to the long-term maintenance of the stem cell phenotype and the rapid proliferation of stem cells in culture. Therefore, we selected above genes as candidates factors based on our hypothesis that such factors may play pivotal roles in MALAT-1 regulated CSCs-like properties. And our results revealed that Sox2 was significantly decreased in response to MALAT-1 knockdown. Sox2, a high-mobility-group DNA binding protein, is a core component of the transcriptional network responsible for maintaining stem cells in a pluripotent, undifferentiated state of self-renewal. Recent studies suggest that Sox2 is aberrantly expressed in pancreatic cancer and involved in later events of carcinogenesis [43], and its overexpression confers pancreatic cancer cell stemness and is sufficient to drive sphere formation and expression of CSC markers [44,45] , as well as induce EMT drivers such as Snail, Twist and Slug [36]. Accumulating evidences have suggested that lncRNAs may act as endogenous sponge RNA to interact with miRNAs and influence the expression of miRNA target genes [46,47]. We performed a search for miRNAs with complementary base paring with MALAT-1 utilizing online software program starbase v2.0 (http://starbase.sysu.edu.cn/mir LncRNA.php) [48], and the results showed that MALAT-1 contains complementary sites to miR-200c and miR-145 (Figure S1). Interestingly, loss of miR-145 elevates Sox2 and impairs differentiation in pancreatic tumors [49]. Lu YX *et al*. [50] found that miR-200c regulated Sox2 expression through a feedback loop and was associated with colorectal carcinoma stemness, growth, and metastasis. Therefore, we hypothesize that MALAT-1 may function as endogenous sponge RNA to interact with miR-200c and miR-145, and upregulate the expression of their target gene Sox2, leading to enhanced stem cell-like phenotypes. But this hypothesis still needs further verification. The detailed mechanism of the regulatory network between MALAT-1 and stem cells related signaling pathway in pancreatic cancer requires further investigation.

## 4. Experimental Section

### 4.1. Cell Culture

Pancreatic cancer cell lines AsPC-1 and CFPAC-1 were purchased from Cell Bank of Chinese Academy of Science. AsPC-1/M-si1 and CFPAC-1/M-si1, which stably knock down expression levels of MALAT-1, as well as that of controls (AsPC-1/M-nc and CFPAC-1/M-nc), were cultured in RPMI-1640 supplemented with 10% fetal bovine serum (FBS, both from Gibco, Carslbad, CA, USA) and 1.5 μg/mL puromycin (Sigma-Aldrich, St. Louis, MO, USA) at 37 °C in a humidified atmosphere of 95% air and 5% CO_2_, and subcultured by harvesting with trypsin-EDTA. Human umbilical vein endothelial cells (HUVEC, Promocell, Heidelberg, Germany) were cultured in same conditions without addition of puromycin. We administered TGF-β1 (5 ng/mL) treatment for a period of 48 h to induced EMT in CFPAC-1 cells.

### 4.2. Real-Time-Quantitative Polymerase Chain Reaction (RT-qPCR) Analysis

RNA was extracted by Trizol reagent method. Reverse transcription in 20 μL system was preformed following protocol of Applied Biosystems. Primers for RT-qPCR were MALAT-1: F-GAATTGCGTCATTTAAAGCCTAGTT, R-GTTTCATCCTACCACTCCCAATTAAT; GAPDH: F-ACAGTCAGCCGCATCTTCTT, R-GACAAGCTTCCCGTTCTCAG. Quantitative mRNA expression was measured by ViiA™ 7 Real-Time PCR System (Applied Biosystems Inc., Foster City, CA, USA). The expression of GAPDH was detected as the endogenous control. Relative mRNA expression of MALAT-1 was calculated with the comparative threshold cycle (*C*_t_) (2^−ΔΔ*C*t^) method.

### 4.3. Soft Agar Assay

To examine the anchorage independent growth, the soft agar assay was performed as follows. Each well of a six-well culture dish was coated in 2× RPMI-1640 with an equal volume of soft agar to give a final solution of 0.6% agar, 1× RPMI-1640, 10% FBS. After the bottom layer solidified, top agar medium mixture (1× RPMI-1640, 10% FBS, 0.3% agar) containing 1 × 10^4^ cells was added, and the dishes were incubated at 37 °C for 4 weeks. Plates were stained with 0.2 mL of MTT (Sigma-Aldrich, 5 mg/mL) for 30 min to identify viable colonies as previous described [51].

### 4.4. Sphere Formation Assay

Sphere formation assay was performed as described elsewhere [52]. In brief, single cell suspensions were washed twice using serum-free Phosphate Buffer Solution (PBS) and plated in 24-well ultralow attachment plates (Corning, Steuben County, New York, NY, USA) at a density of 250 cells in culture media supplemented with 1% N2 supplement (Gibco, Carlsbad, CA, USA), 2% B27 supplement (Gibco, Carlsbad, CA, USA), 20 ng/mL human platelet growth factor (Sigma-Aldrich, St. Louis, MO, USA), 100 ng/mL epidermal growth factor (Gibco, Carlsbad, CA, USA) at 37 °C in a humidified atmosphere of 95% air and 5% CO_2_.

### 4.5. Flow Cytometry Analysis and Cell Sorting

Cells were harvested, disaggregated to a single cell suspension, and stained as described previously. The antibodies PE anti-human CD133 (a dilution of 1:100, Miltenyi Biotech Inc., Bergisch Gladbach, Germany) were added to 1 × 10^6^ cell/100 μL in PBS, and incubated for 20 min on ice in dark. After washing twice with PBS, samples were resuspended in 400 μL PBS and analyzed on a BD Aria II flow cytometer (BD Immunocytometry Systems, San Jose, CA, USA). Forward-scatter and Side-scatter profiles were used to eliminate cell doublets. Positive CD133 sub-populations were identified by comparison of fully-stained samples to FMo (fluorescence-minus-one) controls.

### 4.6. Western Blot Analysis

Cells were washed three times with cold PBS and lysed on ice in RIPA buffer with protease inhibitors PMSF. The protein concentrations were determined using the BCA method (Beyotime Biotechnology, Haimen, China). A total of 30 µg of protein was separated by 10% SDS-PAGE and electro-blotted onto NC membranes using a semi-dry blotting apparatus. After blocking in 3% bovine serum albumin (BSA), the membranes were incubated overnight at 4 °C with the primary antibodies. The membranes were then incubated in the secondary antibodies for 1 h at room temperature on a shaker. The protein bands were visualized by using a commercially available enhanced chemiluminesence kit (Thermo Scientific, Hudson, NH, USA). GAPDH or β-Actin was used as a loading control. Antibodies used for western blot analyses were as follows: Bmi1, Nanog, Oct4 and Sox2 (Cell Signaling Technology, Beverly, MA, USA); c-Myc, β-catenin, β-Actin and GAPDH (Santa Cruz Biotechnology, Santa Cruz, CA, USA); VEGF (Abnova, Taipei, Taiwan).

### 4.7. Chemosensitivity Assay of Gemcitabine

For the chemosensitivity assay, the 50% inhibitory drug concentration (IC_50_) of gemcitabine was determined in M-nc and M-si1 cell lines. Briefly, 5000 cells per well in 96-well plates, in 100 μL of culture medium and cultured overnight. Next day, the medium was supplemented with 0–100 μM gemcitabine (Sigma-Aldrich, St. Louis, MO, USA) for 72 h at 37 °C. Cell proliferation was determined using Sulforhodamine B (SRB) assay. The % cell inhibition was determined using the following formula: % cell inhibition = {100 − [absorbance (sample)/absorbance (control)]} × 100. IC_50_ was determined using GraphPad Prism 6 software (GraphPad, San Diego, CA, USA).

### 4.8. Immunofluorescence

In brief, the cells plated onto poly-l-lysine-coated glass coverslips were fixed with 4% paraformaldehyde, and then washed with PBS. The cells were permeabilized with 0.1% Triton X-100/PBS for 10 min and subsequently incubated with primary antibodies (rabbit monoclonal anti-Sox2 antibody, 1:100, Cell Signaling Technology, Beverly, MA, USA) for detection of specific protein, respectively. Next, the cells were incubated with appropriately fluorescent secondary antibodies at room temperature for 1 h in the dark, followed by incubation with 4',6-Diamidino-2-phenylindole (DAPI) for 5 min before washed three times with PBS to remove excessive staining solution. Samples were imaged with an Olympus IX71 FluoView confocal microscope (Olympus, Tokyo, Japan) with a ×60 oil objective.

### 4.9. VEGF ELISA

Human VEGF Quantikine ELISA Kits (R&D Systems, Minneapolis, MN, USA) was used according to the directions of the manufacturer. Two-hundred microliters of conditioned media were collected from triplicate samples. ELISA values were corrected for total cell protein.

### 4.10. Endothelial Migration Assays and Tube Formation Assays

For endothelial migration assays, conditioned media were harvested from M-nc and M-si1 cells grown in 60-mm plates in RPMI-1640 without growth factors for 24 h. The media were added to the bottom chambers of 24-well tissue culture plates in triplicate. HUVEC (40,000) were added to the upper chambers of Transwell assays (BD Biosciences, Franklin Lakes, NJ, USA). Cells were allowed to migrate for 14 h and then fixed, stained, and quantified.

For endothelial tube formation assays, the above conditioned media were added to 2000 HUVEC in sextuplicate wells of matrigel-coated 96-well plates (BD Biosciences, Franklin Lakes, NJ, USA). The degree of tube formation was evaluated using an inverted microscope. The numbers of tubes were calculated using ImagePro Plus software.

### 4.11. Subcutaneous Nude Mouse Xenografts

Animal studies were conducted in strict accordance with the principles and procedures approved by the Committee on the Ethics of Animal Experiments of Shanghai Jiao Tong University School of Medicine. Six 4-week-old female BALB/c nude mice (Institute of Zoology, China Academy of Sciences) were randomly divided into two groups (3 for each group). 2 × 10^6^ cell numbers of CFPAC-1/M-nc or CFPAC-1/M-si1 in 100 μL PBS/Matrigel (75:25) were inoculated subcutaneously. Tumor nodules were measured every 7 days up to 4 weeks, and the volume of a tumor was calculated by following formula: Volume (mm^3^) = (A × B^2^)/2, where A is the longest diameter of tumor and B is the shortest diameter. 

### 4.12. Immunohistochemistry 

Immunohistochemistry was performed on formalin-fixed paraffin-embedded sections. The sections were dewaxed and dehydrated. After rehydration, endogenous peroxidase activity was blocked for 15 min with 0.3% hydrogen peroxide. After antigen retrieval in citrate buffer, the sections were blocked with 3% BSA, and then probed with anti-Ki67 (Cell Signaling Technology, Beverly, MA, USA) and anti-CD31 antibody at 4 °C overnight (Abcam Inc., Cambridge, MA, USA), respectively, followed incubated by HRP labeled secondary antibody and were visualized by diaminobenzidine.

### 4.13. Statistical Analysis

Data were presented as mean ± SD unless otherwise indicated of at least three independent experiments. Statistical analysis was performed using a SPSS 13.0 package system. Statistical significance was assessed by the Student’s *t*-test. A *p* value less than 0.05 was considered statistically significant.

## 5. Conclusions

In the present study, we revealed that MALAT-1 could increase the proportion of pancreatic CSCs, maintain self-renewing capacity, decrease the chemosensitivity to anticancer drugs, and accelerate tumor angiogenesis *in vitro*, and promote tumorigenicity of pancreatic cancer cells *in vivo*. The underlying mechanisms may involve in increased expression of self-renewal related factors Sox2. In conclusion, our finding suggests a novel role of MALAT-1 that could enhance stem cell-like phenotypes in pancreatic cancer cells, which remains to be fully elucidated.

## Supplementary Materials

Supplementary materials can be found at http://www.mdpi.com/1422-0067/16/04/6677/s1.
