# Supplementary material for: Long Noncoding RNA MALAT-1 Enhances Stem Cell-Like Phenotypes in Pancreatic Cancer Cells
Source: Int J Mol Sci. 2015 Mar 24;16(4):6677–93. doi: 10.3390/ijms16046677 (PMC4424983; doi:10.3390/ijms16046677)
Supplement: Supplementary file 1 [file ijms-16-06677-s001.pdf]

Supplementary Information

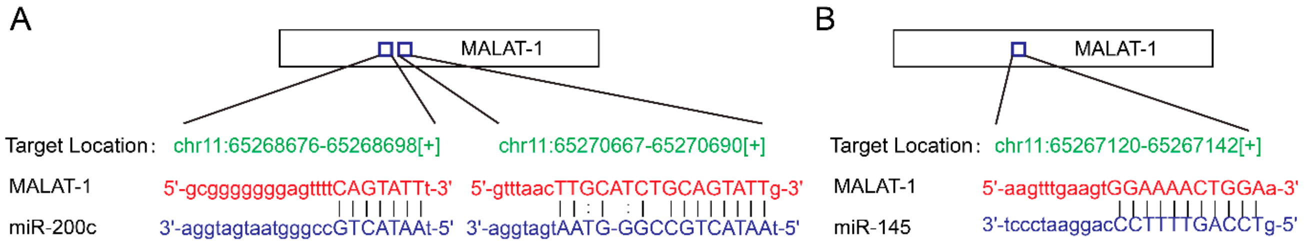

Figure S1. The potential MALAT-1 base pairing with miR-200c (**A**) and miR-145 (**B**) as identified by Starbase v2.0 (http://starbase.sysu.edu.cn/mirLncRNA.php).
